# Burden of headaches, eye irritation and respiratory symptoms among females stacking LPG with polluting cooking fuels: Modelling from peri-urban Cameroon, Ghana & Kenya

**DOI:** 10.1016/j.nexus.2024.100304

**Published:** 2024-07

**Authors:** Kourosh Parvizi, Diana Menya, Emily Nix, Judith Mangeni, Federico Lorenzetti, Edna Sang, Rachel Anderson de Cuevas, Theresa Tawiah, Miranda Baame, Emmanuel Betang, Sara Ronzi, Mieks Twumasi, Seeba Amenga-Etego, Reginald Quansah, Bertrand Hugo Mbatchou Ngahane, Elisa Puzzolo, Kwaku Poku Asante, Daniel Pope, Matthew Shupler

**Affiliations:** a Department of Public Health, Policy and Systems, University of Liverpool, United Kingdom; b School of Public Health, Moi University, Eldoret, Kenya; c Kintampo Health Research Centre, Kintampo, Ghana; d Douala General Hospital, Douala, Cameroon; e School of Public Health, University of Ghana, Ghana

**Keywords:** LPG, Health symptoms, Respiratory, Fuel stacking, Clean cooking, Sub-Saharan Africa

## Abstract

**Introduction:**

Liquefied petroleum gas (LPG) is a clean cooking fuel that emits less household air pollution (HAP) than polluting cooking fuels (e.g. charcoal, wood). While switching from polluting fuels to LPG can reduce HAP and improve health, the impact of ‘stacking’ (concurrent use of polluting fuels and LPG) on adverse health symptoms (e.g. headaches, eye irritation, cough) among female cooks is uncertain.

**Methods:**

Survey data from the CLEAN-Air(Africa) study was collected on cooking patterns and health symptoms over the last 12 months (cough, wheezing, chest tightness, shortness of breath, eye irritation, headaches) from approximately 400 female primary cooks in each of three peri‑urban communities in sub-Saharan Africa: Mbalmayo, Cameroon; Obuasi, Ghana; and Eldoret, Kenya. Random effects Poisson regression, adjusted for socioeconomic and health-related covariates, assessed the relationship between primary and secondary cooking fuel type and self-reported health symptoms.

**Results:**

Among 1,147 participants, 10 % (*n* = 118) exclusively cooked with LPG, 45 % (*n* = 509) stacked LPG and polluting fuels and 45 % (*n* = 520) exclusively cooked with polluting fuels. Female cooks stacking LPG and polluting fuels had significantly higher odds of shortness of breath (OR 2.16, 95 %CI:1.04–4.48) compared with those exclusively using LPG. In two communities, headache prevalence was 30 % higher among women stacking LPG with polluting fuels (Mbalmayo:82 %; Eldoret:65 %) compared with those exclusively using LPG (Mbalmayo:53 %; Eldoret:33 %). Women stacking LPG and polluting fuels (OR 2.45, 95 %CI:1.29–4.67) had significantly higher odds of eye irritation than women cooking exclusively with LPG. Second-hand smoke exposure was significantly associated with higher odds of chest tightness (OR 1.92, 95 % CI:1.19–3.11), wheezing (OR 1.76, 95 % CI:1.06–2.91) and cough (OR 1.78, 95 %CI:1.13–2.80).

**Conclusions:**

In peri‑urban sub-Saharan Africa, women exclusively cooking with LPG had lower odds of several health symptoms than those stacking LPG and polluting fuels. Promoting a complete transition to LPG in these communities may likely generate short-term health benefits for primary cooks.

## Highlights

• Women stacking LPG & polluting fuels had higher shortness of breath odds than LPG-only users.
• Headache prevalence 30 % higher among women stacking LPG & polluting fuel than using only LPG.
• Women stacking LPG and polluting fuels had higher odds of eye irritation than exclusive LPG users.
• Second-hand smoke exposure associated with higher odds of chest tightness, wheezing & cough.
• Complete transition to LPG can likely generate short-term health benefits for primary cooks.

## 1 Introduction

### 1.1 The global burden of household air pollution

Approximately 3.2 million deaths, predominantly in low- and middle-income countries (LMICs [1]), were attributed to household air pollution (HAP) exposure from cooking, heating and lighting with polluting fuels (e.g. kerosene, wood, charcoal) in 2019 [[2], [3], [4]]. Multiple studies have established a causal relationship between exposure to components of HAP, such as carbon monoxide (CO) and fine particulate matter (PM_2.5_) with several diseases of the respiratory system (childhood pneumonia, chronic obstructive pulmonary disease (COPD), and lung cancer) [[5], [6], [7], [8], [9], [10], [11]].

In addition to various adverse respiratory outcomes, HAP exposure has been associated with shorter-term respiratory symptoms such as wheezing, cough and excess phlegm in the throat [12]. Exposure to smoke from cooking with polluting fuels can also lead to eye irritation [13] and breathing in CO from burning biomass can cause headaches [14].

### 1.2 Reducing the negative health effects of household air pollution

Reducing the health burden of HAP exposure requires expanded access to and adoption of cleaner fuels [15], such as liquified petroleum gas (LPG), electricity, biogas and alcohol fuels, that generate no or low levels of health-damaging HAP [16,17]. In sub-Saharan Africa (SSA), where 85 % of individuals cook with polluting fuels [18], LPG is a more scalable clean fuel than electricity for short- to medium-term expansion as it requires less infrastructure [19,20].

Transitioning to LPG can also benefit the environment and climate through reductions in localized deforestation (from unsustainable harvesting of wood fuel or for charcoal production) [21] and in emissions of black carbon relative to combustion of polluting fuels. The potential for LPG to reduce HAP emissions and benefit the climate through rapid scale [22] has prompted some SSA countries (e.g. Cameroon, Rwanda and Kenya) to develop plans for substantially increasing the population proportion using LPG for cooking by 2030 [[23], [24], [25]].

### 1.3 The “Stacking” phenomenon

When households switch from polluting fuels to LPG, the transition usually is not seamless. Rather, families will continue to use their polluting fuel alongside LPG to meet all of their cooking needs [26]. This phenomenon, called fuel ‘stacking’, is highly prevalent in LMICs; studies have reported a stacking prevalence ranging from around 30–100 % [26,27]. Stacking results from many reasons, including affordability, cultural compatibility, stove functionality, household dynamics, safety issues, fuel supply issues, technical characteristics, and time aspects. For example, preference for the taste of foods on traditional stoves or and the desire not to pay for large quantities of gas for certain meals that require a longer cooking time (e.g. stews) can increase the use of polluting fuels [26,28,29]. Additionally, variation in seasonal needs or changes in fuel prices can cause households to stack multiple fuels [30,31].

### 1.4 Aims and objectives

Although the pervasiveness of fuel stacking has been well documented, there are minimal studies directly investigating the impact of fuel stacking versus exclusive clean fuel use on short-term health symptoms (e.g. coughing, wheezing, headache, eye irritation). Studies that have been conducted in SSA have *mostly* examined the association between respiratory health symptoms prevalence and primary cooking fuel type only [[32], [33], [34]]. These studies have found that cooking with a primary polluting fuel was associated with increased odds of respiratory symptoms, such has cough, wheeze and difficulty breathing, among female primary cooks [33,34] and respiratory outcomes, like pneumonia, among children [32]. As stacking is known to prevent reductions in PM_2.5_ levels below WHO Indoor Air Quality guidelines that are needed to achieve greater health benefits, [30,[35], [36], [37]] understanding how fuel stacking affects the reporting of respiratory health symptoms among primary cooks is important to document [12,38].

This study therefore investigates the association between fuel stacking and adverse health symptoms among female primary cooks (individuals responsible for the majority of cooking in the household) living in peri‑urban communities in three SSA countries (Cameroon, Ghana, and Kenya). The prevalence of respiratory symptoms (cough, wheezing, chest tightness, shortness of breath), eye irritation, and headaches was modelled in relation to primary and secondary cooking fuel combinations (households using polluting fuels exclusively, stacking polluting fuels and LPG, and using LPG exclusively). Such information can uncover potential health implications of an incomplete transition to LPG for cooking.

## 2 Methods

### 2.1 Study setting and population

*The CLEAN-Air(Africa)* study consisted of three phases (census survey, in-depth survey, HAP measurements) to evaluate impacts of fuel use and cooking practices on household air pollution and health amongst peri‑urban communities in Cameroon (Mbalmayo, central region), Ghana (Obuasi, Ashanti region) and Kenya (Eldoret, Western Kenya) [39]. Mblamayo (population: 70,000) is an agricultural town near Yaoundé (Cameroon's Capital). Obuasi (population: 200,000) is a gold-mining town close to Kumasi (capital of the Ashanti region, Ghana). Eldoret (population: 500,000) is an agricultural town in Western Kenya.

The first data collection phase involved a 20 min census survey administered to approximately 2000 randomly selected households from each of the three study communities (6000 participants in total) via door-to-door sampling. The survey assessed household demographics, socio-economic status, and current domestic fuel/stove use [40]. For 1–2 weeks before data collection began, residents underwent a period of engagement and sensitisation; community workers communicated the research aims, how the data would be collected and how it may impact households.

The current study presents data from the second phase of the study, consisting of more detailed in-depth surveys (∼40 min) that measured cooking and health behaviours (e.g., smoking/alcohol use) and health symptoms deemed relevant to HAP exposure, including respiratory symptoms (cough, wheezing, chest tightness, shortness of breath), eye irritation, and headaches. Cooking-related survey questions were based on the WHO harmonized household energy survey questions [41].

**Fig. 1:**
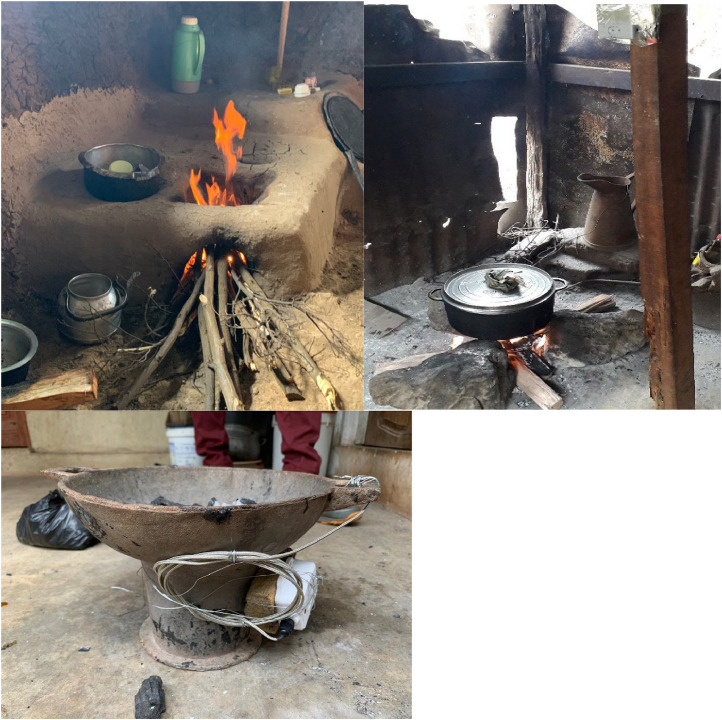
Common polluting fuel stoves used in Eldoret, Kenya (chepkube mud stove - top left), Mbalmayo, Cameroon (three stone fire – top right) and Obuasi, Ghana (manufactured charcoal stove – bottom).

Stratified random sampling by primary cooking fuel type reported in the census survey (phase 1) was used to select households for phase 2 ensuring roughly equal numbers of households cooking primarily with LPG and exclusively with polluting fuels. Polluting fuel stoves used in the study communities were predominantly open fires in Mbalmayo, charcoal stoves in Obuasi, and chepkube (locally made from mud) stoves in Eldoret (Fig. 1). The polluting fuel stoves used by study participants did not have a chimney.

**Fig. 2:**
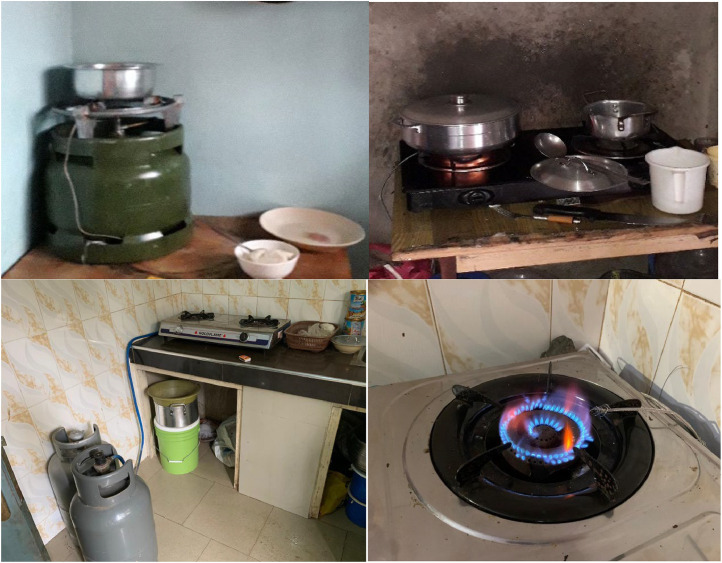
Common LPG stoves used in Eldoret, Kenya (single burner (Meko) stove – top left), Mbalmayo, Cameroon (double burner stove – top right) and Obuasi, Ghana (double burner stove – bottom left and right).

LPG stoves used in the study communities were mostly double burner stoves in Mbalmayo and Obuasi, and single burner (Meko) stoves in Eldoret (Fig. 2).

Phase 2 surveys were completed by approximately 200 households primarily using LPG and 200 households exclusively using polluting cooking fuels in each of the three communities (total of 1200 households). After excluding smokers (*n* = 12, 1 %), males (*n* = 49, 4 %), those that were not the main household cook (*n* = 3, 0.2 %), responders who listed electricity as a secondary fuel (*n* = 3, 0.2 %) and those with incomplete entries for cooking fuel type (*n* = 20, 1.6 %) due to small sample size, the final analytic sample consisted of 1147 primary cooks (93 % of initial sample).

The surveys were administered by trained fieldworkers using the Mobenzi Researcher secure digital data collection application [42] on secure mobile phones. Data entered via the Mobenzi App were uploaded via the phones SIM (or via Wi-Fi in areas where there is no reception) to the Mobenzi cloud (all data encrypted at source).

### 2.2 Outcome variables

Outcome variables included several standardised self-reported adverse respiratory symptoms: cough (any of the following types: night time, first thing in the morning, with phlegm or, most mornings or more than three months per year), wheezing (in the last 12 months), chest tightness (in the last 12 months), headaches (in the last 12 months), eye irritation (whilst cooking), and shortness of breath (any of the following types: during strenuous activity, not during strenuous activity, night-time attacks). Full survey questions are included in Supplementary Table 1.

### 2.3 Explanatory variables

The main explanatory variables were designed to assess fuel stacking based upon participant responses regarding their primary and secondary cooking fuels (e.g. wood, charcoal, kerosene, LPG, etc.). Participant answers for primary or secondary fuel type were grouped into either “LPG” or “polluting fuel” categories.

For models assessing ‘stacking’ as the main exposure, households that reported cooking with LPG as their primary fuel and a polluting secondary fuel, or vice versa, were labeled as ‘stacking’, while those exclusively cooking with LPG or with polluting fuels were grouped into “LPG exclusive” or “polluting fuel exclusive” categories (Table 1). Responders who listed electricity as a secondary fuel were removed due to small sample size (*n* = 3, 0.2 %). Incomplete entries for cooking fuel (*n* = 20, 1.6 %) were also removed. The final sample size was 1147 primary cooks (93 % of initial sample).

**Table 1:** Categorization of exposure variables for Poisson regression.

Explanatory variable	Description	Categories	Primary fuel type	Secondary fuel type
Primary fuel	What is the primary fuel used for cooking?	LPG^1^	LPG	*Not considered*
		Polluting	Polluting	
Stacking	Is LPG/Polluting fuel used exclusively across primary and secondary fuel or is there mixing (stacking)?	LPG exclusive^1^	LPG	LPG / no secondary fuel used
		Polluting exclusive	Polluting	Polluting / no secondary fuel used
		Stacking	LPG	Polluting
			Polluting	LPG

1 = Reference group used in analysis.

In study communities with less than two-thirds of households having electricity access for lighting (to ensure sufficient power to detect differences by electricity status), symptom prevalence by the existence of an electricity connection was also examined descriptively.

To assess whether the relative percent of cooking with LPG affects the prevalence of self-reported outcomes, the proportion of LPG use was estimated from a survey question that asked, “*On a typical weekday (*e.g. *Monday or Tuesday), please estimate how much time you spend cooking with (i) your main/ primary stove/ fuel and (ii) your second most common stove/fuel?*”. As a proxy measure of the proportion of cooking done with LPG, the self-reported time spent cooking with LPG on a weekday was divided by the total time spent cooking with all cooking fuels on a weekday. Households in the ‘stacking’ group (Table 1) were split into two equal groups of those that reported using LPG for at least two-thirds (65–99 %) of their cooking on weekdays and those than used LPG for less than two-thirds of their weekday cooking (1–65 %). Sensitivity analyses were conducted by including these stacking groups (1–65 % vs 66–99 %) in additional models for each health outcome.

### 2.4 Covariates

Covariates considered in all models included age, education level, financial security (“Do you have enough money to meet your household needs?” “Enough”, “Not quite enough”, “Definitely not enough”), presence of a diagnosed chronic health condition (tuberculosis, high blood pressure or heart disease) [Supplementary Table 1] and access to an electricity connection (for lighting or other non-cooking tasks). Financial security was used in place of household income due to a large number of non-responses to a question about income level (*n* = 266, 22 %). All variables were categorical except for age, which was continuous.

Exposure to second-hand smoke was also included in the models. We asked the following questions to all respondents: “Does anyone in your household smoke cigarettes?” Followed by the question “If Yes, do they smoke in the house, or only outside the house?”. Participants that responded ‘yes’ to the first question and ‘in the house’ to the second question we classified as having second-hand smoke exposure.

### 2.5 Statistical analysis

Separate multivariable random effects (community-level random intercept) Poisson regression models were built to assess the relationship between cooking fuel use and each self-reported respiratory health symptom (binary: yes/no). Poisson regression was used in place of logistic regression to avoid overestimating the odds ratio due to the high prevalence of several health symptoms reported by the study population [43,44].

Model selection was based on optimising the Akaike Information Criterion (AIC), and coefficient of determination (R^2^) [Supplementary Table 3]. The intraclass correlation coefficient (ICC) was examined in all models to assess the intra-community level variation in the associations. Model coefficients are presented as odds ratios (OR) with 95 % confidence intervals (CI). Data analysis was carried out in R version 4.0.3 [45].

## 3 Results

### 3.1 Demographics

The final analytic sample contained 1147 female primary cooks (Mbalmayo *n* = 403, 35.1 %; Obuasi *n* = 347, 30.3 %; Eldoret *n* = 397, 34.6 %). The mean age of participants was 35.3 years (SD 11.6) and comparable between countries (Mbalmayo 35.7, SD 12.7; Obuasi 35.5, SD 10.3; Eldoret 34.9, SD 11.5) (Table 2). Most participants were married (*n* = 648, 56.5 %). The majority had received a secondary school education (*n* = 605, 52.7 %), except in the Kenyan community, where the most common education level was university (*n* = 138, 34.8 %).

**Table 2:** Demographics characteristics of study population.

	Total (*n* = 1147)	Mbalmayo (*n* = 403)	Obuasi (*n* = 347)	Eldoret (*n* = 397)
**Age, mean (SD)**	35.3 (11.6)	35.7 (12.7)	35.5 (10.2)	34.9 (11.5)
**Marital status, n (%)**				
Divorced/separated	26 (2.3)	10 (2.5)	9 (2.6)	7 (1.8)
Living together	152 (13.3)	108 (26.8)	44 (12.7)	0 (0)
Married	648 (56.5)	160 (39.7)	212 (61.1)	276 (69.5)
Single	253 (22.1)	100 (24.8)	62 (17.9)	91 (22.9)
Widow	68 (5.9)	25 (6.2)	20 (5.8)	23 (5.8)
**Education level, n (%)**				
No formal education	58 (5.1)	5 (1.3)	39 (11.2)	14 (3.5)
Primary school	297 (25.9)	105 (27.5)	71 (20.5)	121 (30.5)
Secondary school	605 (52.7)	237 (61.9)	224 (64.6)	124 (31.2)
University	187 (16.3)	36 (9.4)	138 (3.7)	138 (34.8)
**Housing status, n (%)**				
Own	499 (45.7)	167 (42.0)	98 (32.3)	234 (59.8)
Rent	593 (54.3)	231 (58.0)	205 (67.7)	157 (40.2)
Other	55 (5.0)	5 (1.3)	44 (14.5)	6 (1.5)
Financial security*, n (%)				
Enough money	264 (23.0)	39 (9.7)	132 (38.0)	93 (23.4)
Not quite enough money	571 (49.8)	199 (49.8)	138 (39.8)	234 (58.9)
Definitely not enough money	312 (27.2)	165 (27.2)	77 (22.2)	70 (17.6)
**Electricity connection, n (%)**				
Yes	836 (72.9)	238 (59.1)	325 (93.7)	273 (68.8)
No **Primary lighting fuel, n (%)** Electricity (inc solar panels) Solar powered lantern/flashlight Battery powered flashlight Rechargeable flashlight Oil lamp Candle **Cooking location** Main house; separate room Main house; no separate room Veranda or covered porch Outside: separate room Outside: open air Other	311 (27.1) 1071 (93.4) 54 (4.7) 2 (0.2) 6 (0.5) 11 (1.0) 3 (0.3) 337 (29.4) 110 (9.6) 255 (22.2) 267 (23.3) 118 (10.3) 60 (5.2)	165 (40.9) 401 (99.5) 0 (0) 0 (0) 0 (0) 1 (0.2) 1 (0.2) 85 (21.1) 41 (10.2) 30 (7.4) 116 (28.8) 72 (17.9) 59 (14.6)	22 (6.3) 341 (98.3) 0 (0) 2 (0.6) 4 (1.2) 0 (0) 0(0) 61 (17.6) 10 (2.9) 224 (64.6) 12 (3.5) 40 (11.5) 0 (0)	124 (31.2) 329 (82.9) 54 (13.6) 0 (0) 2 (0.5) 10 (2.5) 0 (0) 191 (48.1) 59 (14.9) 1 (0.3) 139 (35.0) 6 (1.5) 1 (0.3)
**Diagnosed with chronic condition**⁎⁎**, n (%)**				
Yes	164 (14.3)	65 (16.1)	44 (12.7)	55 (13.9)
No	983 (85.7)	338 (83.9)	303 (87.3)	342 (86.1)
**Other smokers in household, n (%)**				
Yes	69 (6.0)	37 (9.2)	15 (4.3)	17 (4.3)
No	1078 (94.0)	366 (90.8)	332 (95.7)	380 (95.7)
**Primary fuel, n (%)**				
LPG	526 (45.9)	178 (44.2)	167 (48.1)	181 (45.6)
Polluting	621 (54.1)	225 (55.8)	180 (51.9)	216 (54.4)
**Secondary fuel, n (%)**				
LPG	192 (16.7)	67 (16.6)	61 (17.6)	64 (16.1)
Polluting	826 (72.0)	303 (75.2)	190 (54.8)	333 (83.9)
No secondary fuel	129 (11.2)	33 (8.2)	96 (27.7)	0 (0)
**Stacking behaviour, n (%)**				
LPG exclusive	118 (10.3)	17 (2.8)	68 (14.7)	33 (5.7)
Polluting fuel exclusive	520 (45.3)	172 (27.9)	163 (35.2)	185 (32.1)
Stack LPG and polluting fuel	509 (44.4)	214 (34.7)	116 (25.1)	179 (31.1)
*LPG as primary fuel*	408 (35.6)	161 (26.1)	99 (21.4)	148 (25.7)
*Polluting fuel as primary fuel*	101 (8.8)	53 (8.6)	17 (3.7)	31 (5.4)

⁎ Financial security assessed with survey question “Do you feel you have enough money available for your weekly required spend”.

⁎⁎ Chronic conditions included Tuberculosis, Heart Disease, Hypertension and Chronic Bronchitis.

Whilst half the surveyed cooks rated their own financial status as “Not quite enough money to buy everything needed” (*n* = 571, 49.8 %;), the proportion of financially secure individuals was substantially larger in Obuasi (*n* = 132, 38.0 %) compared with Mbalmayo (*n* = 39, 9.7 %) and Eldoret (*n* = 93, 23.4 %) (Table 2). Most respondents had an electricity connection (*n* = 836, 72.9 %), did not have a chronic health condition (*n* = 983, 85.7 %) and did not live with smokers (*n* = 1078, 94.0 %) (Table 2).

### 3.2 Primary and secondary fuel choice

Stratified randomisation for phase 2 surveys resulted in a roughly even percentage of participants primarily using LPG and or polluting fuels (polluting fuels: *n* = 621, 54.1 %; LPG: *n* = 520, 45.9 %). Polluting cooking fuels used in the three study communities were almost exclusively wood (Mbalmayo, Eldoret) or charcoal (Obuasi); the type of wood stove varied by community, with three stone fires being the most common in peri‑urban Cameroon and Ghana and a locally manufactured ‘chepkube’ mud stove (used indoors) being most frequently used in the Kenyan community.

Most participants stacked multiple fuels (*n* = 1018; 88.8 %); only 10 % of households reported exclusively cooking with LPG (*n* = 118). Exclusive LPG users were more common in Obuasi (*n* = 68, 14.7 %) than in Eldoret (*n* = 33; 5.7 %) and Mbalmayo (*n* = 17, 2.8 %) (Table 2).

In Mbalmayo, the main cooking location was outside in a separate room (28.8 %) or open air (17.9 %), whilst in Obuasi it was primarily on a veranda/covered porch (64.6 %). Participants in Eldoret mostly cooked in a separate room in the main house (48.1 %). The primary lighting fuel amongst all three communities was electricity (Mbalmayo 99.5 %; Obuasi 98.3 %; Eldoret 82.9 %); in Eldoret, a small proportion of participants used solar power lanterns or flashlights as their main lighting source (13.6 %) (Table 2).

### 3.3 Prevalence of health symptoms by cooking fuel type

**Fig. 3:**
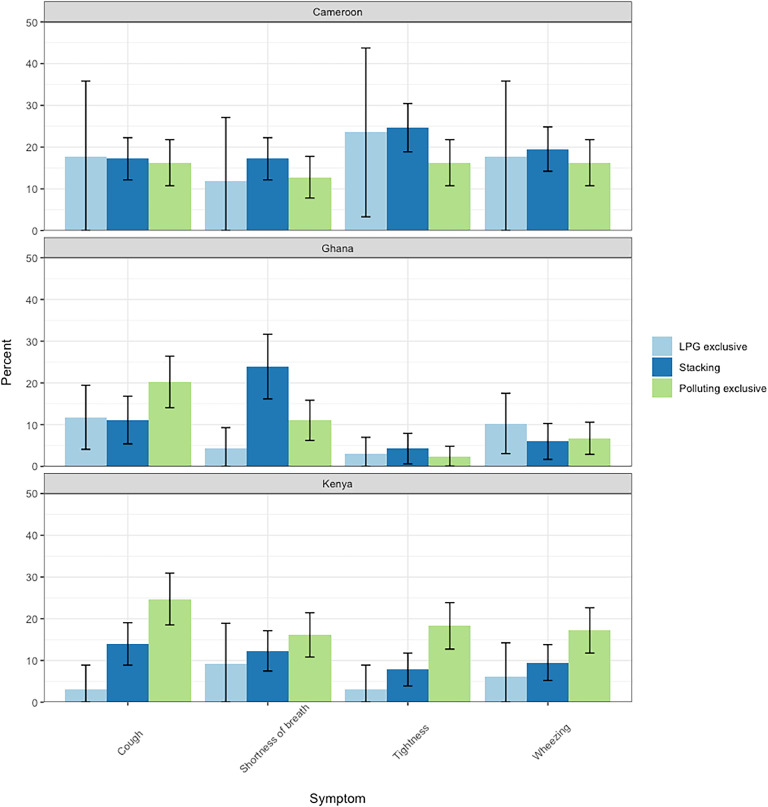
Prevalence of respiratory symptoms in whole study population, stratified by community and stacking behaviour.

Two-thirds of participants reported having headaches (*n* = 745, 65.0 %) and one-third reported having eye irritation (*n* = 378, 33.0 %) (Fig. 3). Nearly all health symptoms were more prevalent in Mbalmayo than in Eldoret and Obuasi (Supplementary Table 2). The prevalence of non-respiratory outcomes varied significantly by stacking behaviour; in Mbalmayo, eye irritation was twice as high among women stacking LPG with a polluting fuel (59.5 %) and exclusively using polluting fuels (63.4 %) than among those cooking with LPG exclusively (23.5 %). Eye irritation in Eldoret was non-existent (0 %) among LPG exclusive users and monotonically increased amongst those who stacked (16.2 %) and those using polluting fuel exclusively (32.3 %). In Obuasi, the prevalence of eye irritation among women exclusively cooking with polluting fuels (17.8 %) was double that among cooks only cooking with LPG (8.8 %).

In Eldoret, the prevalence of headaches was twice as high amongst female cooks who stacked LPG with a polluting fuel (64.8 %) and those using polluting fuels exclusively (61.8 %) compared with cooks using LPG exclusively (33.3 %) (Supplementary Fig. 1). In Mbalmayo, headaches were more common among women stacking LPG with a polluting fuel (81.8 %) and cooking exclusively with polluting fuels (72.1 %) compared with LPG exclusive users (52.9 %). There were no significant differences in headache prevalence by cooking fuel type in Obuasi.

Symptoms of cough (*n* = 194, 16.9 %), shortness of breath (*n* = 165, 14.4 %), wheezing (*n* = 149, 13.0 %) and chest tightness (*n* = 145, 12.6 %) were less prevalent than non-respiratory symptoms (Fig. 3). While no differences in reporting of cough was detected by cooking fuels used in Mbalmayo, a statistically significant difference was detected in Eldoret; LPG exclusive users had a significantly lower prevalence of cough (3.0 %) than those stacking LPG and polluting fuels (14.0 %) and exclusively cooking with polluting fuels (24.7 %). While not reaching statistical significance in Obuasi, the prevalence of cough was nearly twice as high among women cooking exclusively with polluting fuels (20.2 %) as those who stacked fuels (11.1 %) or used LPG exclusively (11.8 %).

Similarly, there was no significant difference in reporting of chest tightness among women cooking with LPG exclusively, stacking fuels and only using polluting fuels in Mbalmayo and Obuasi. Chest tightness was however statistically significantly higher among those using polluting fuels exclusively (18.3 %) compared with those who used LPG exclusively (3.0 %) in Eldoret.

In all three communities, the prevalence of shortness of breath was greater among women stacking LPG and polluting fuels (Mblamyo = 17.3 %, Obuasi = 29.3 % Eldoret = 12.3 %), compared to those exclusively cooking with LPG (Mblamyo = 11.8 %, Obuasi = 4.4 %, Eldoret = 9.1 %). In Eldoret, prevalence of shortness of breath amongst exclusive polluting fuel users (16.1 %) was also higher than among women stacking LPG and polluting fuels.

### 3.4 Prevalence of health symptoms by electricity access

**Fig. 4:**
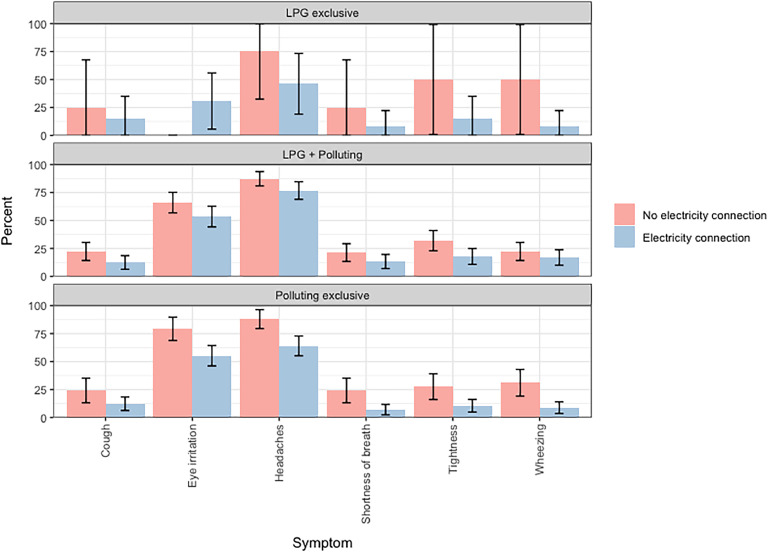
Prevalence of recorded symptoms in Mblamayo, Cameroon stratified by electricity access and stacking behaviour.

Mbalmayo was the only community with less than 65 % of the population having an electricity connection for lighting (Table 2). The prevalence of respiratory symptoms was consistently higher among women without electricity access compared to those with access in Mbalmayo, irrespective of the type of cooking fuel used (Fig. 3). Among women exclusively using polluting fuels in Mbalmayo, the prevalence of several health symptoms, including eye irritation, headaches, shortness of breath and wheezing, was statistically significantly higher among those without access to electricity compared to those with access (Fig. 4).

### 3.5 Modelling results

#### 3.5.1 Wheezing

Women stacking LPG with a polluting fuel or using polluting fuels exclusively (Supplementary Table 5) did not have higher odds of wheezing than those exclusively cooking with LPG. However, exposure to second-hand tobacco smoke was significantly associated with an increased odds (OR 1.76, 95 % CI 1.06–2.91) of wheezing (Supplementary Fig. 2), as was lack of an electricity connection (OR 1.87, 95 % CI 1.29–2.71) (Supplementary Fig. 2).

#### 3.5.2 Chest tightness

Female primary cooks cooking exclusively with polluting fuels (OR: 1.21, 95 % CI 0.55–2.66) or stacking LPG and a polluting cooking fuel (OR: 1.33, 95 % CI 0.61–2.93) did not have significantly higher odds of chest tightness compared with those exclusively cooking with LPG [Supplementary Table 7]. However, exposure to second-hand tobacco smoke (OR 1.92, 95 % CI 1.19 – 3.11), a diagnosis of a chronic condition (OR 2.01, 95 % CI 1.35 – 3.01), and lack of access to electricity for lighting (OR 1.69, 95 % CI 1.69 – 2.37) were all significantly associated with increased odds of chest tightness amongst primary cooks (Supplementary Fig. 3).

#### 3.5.3 Shortness of breath

Women stacking LPG and polluting fuels had significantly increased odds of shortness of breath (OR 2.16, 95 % CI 1.04–4.48) compared with women exclusively cooking with LPG (Supplementary Table 9). Women cooking exclusively with polluting fuels had an elevated odds of shortness of breath (OR 1.57, 95 % CI 0.75–3.30) compared with women exclusively cooking with LPG.

Additionally, women reporting financial insecurity (“Not quite financially secure” - OR: 1.72, 95 % CI 1.12–2.64), the presence of a chronic condition (OR 2.05, 95 % CI 1.41–2.98), and a lack of access to electricity connection (OR 1.69, 95 % CI 1.23–2.33) had significantly increased odds of shortness of breath (Supplementary Fig. 4).

#### 3.5.4 Cough

Women cooking exclusively with polluting fuels (OR 1.65, 95 % 0.90–3.02) and stacking LPG with a polluting fuel (OR 1.29, 95 % CI 0.70–2.39) had an elevated odds of cough compared to LPG exclusive users, although not statically significant (Supplementary Table 11). Financial insecurity (“Not quite financially secure” – OR 1.56, 95 % CI 1.05–2.32), second-hand tobacco smoke exposure (OR 1.78, 95 % CI 1.13–2.80), diagnosis of a chronic health condition (OR 1.79, 95 % CI 1.25–2.57) and lack of electricity connection (OR 1.40, 95 % CI 1.04–1.89) were all also associated with an increased odds of cough (Supplementary Fig. 5).

#### 3.5.5 Eye irritation

**Fig. 5:**
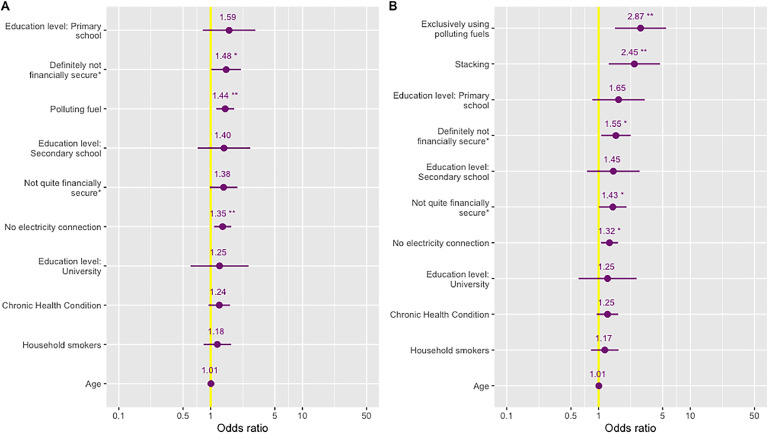
Odds (95 % CI) of eye irritation for primary fuel model (A) and stacking model (B) * Not quite financially secure represents individuals reporting “Not quite enough money” to live. ** Definitely not financially secure represents individuals reporting “Definitely not enough money” to live.

Women cooking exclusively with polluting fuels (OR 2.87, 95 % CI 1.51–5.45) or stacking LPG with a polluting fuel (OR 2.45, 95 % CI 1.29–4.67) had over twice the odds of eye irritation as women exclusively cooking with LPG (Supplementary Table 14, Fig. 5). Women reporting financial insecurity (“Definitely not secure”, OR 1.48, 95 % CI 1.02–2.14) and not having an electricity connection (OR 1.35, 95 % CI 1.09–1.67) also had a significantly increased odds of eye irritation (Fig. 5).

#### 3.5.6 Headaches

**Fig. 6:**
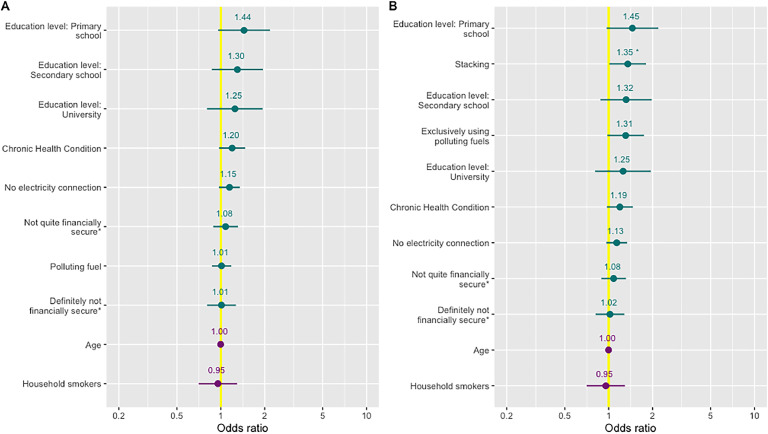
Odds (95 % CI) of headache for primary fuel model (A) and stacking model (B) * Not quite financially secure represents individuals reporting “Not quite enough money” to live. ** Definitely not financially secure represents individuals reporting “Definitely not enough money” to live.

There was no difference in the odds of headaches among women primarily cooking with LPG and primary using a polluting cooking fuel (OR 1.01 95 % CI 0.87 – 1.18). However, female cooks exclusively using polluting cooking fuels had marginally significantly increased odds of headaches (OR 1.31, 95 % CI 0.98–1.75) compared with exclusive LPG users (Supplementary Table 15, Fig. 6). Furthermore, cooks stacking LPG and polluting fuels had significantly increased odds (OR 1.35, 95 % CI 1.01–1.80) of headache compared to women exclusively using LPG. All other covariates in the model were not significantly associated with increased odds of headache (Supplementary Table 15, Fig. 6).

#### 3.5.7 Effect of relative time spent cooking with LPG on health symptoms

The sensitivity analysis assessing the effect of relative time spent cooking with LPG on measured health symptoms revealed no significantly reduced odds of shortness of breath, wheezing, chest tightness or headaches amongst those who used LPG for 1–64 %, 65–99 % or 100 % of their cooking compared to those who used polluting fuels exclusively (Supplementary Tables 17–20).

Eye irritation prevalence was reduced amongst those using LPG for 100 % of the time spent cooking [OR 0.43, 95 % CI 0.26–0.72]. The protective effect monotonically weakened among those using LPG for 65–99.9 % [OR 0.74, 95 % CI 0.52–1.04] or 1–64 % [OR 0.86, 95 % CI 0.64–1.15] of their cooking time (Supplementary Table 21). There was some evidence of a monotonically increasing association between greater proportional use of LPG and reduced odds of coughing (1–64 %: OR 0.82, 95 % CI 0.53–1.26; 65–99 %: OR 0.77, 95 % CI 0.48–1.25; 100 %: OR 0.61, 95 % CI 0.35–1.06) (Supplementary Table 16).

## 4 Discussion

In one of the first studies to assess the impact of cooking fuel stacking on health symptoms, stacking LPG with polluting fuels (charcoal or firewood) or cooking only with polluting fuels as opposed to exclusive use of LPG was associated with increased odds of multiple adverse health symptoms, including shortness of breath, headaches and eye irritation, among female primary cooks living in peri‑urban communities of sub-Saharan Africa. With significantly lower odds of shortness of breath and marginally significantly lower odds of cough among exclusive versus primary LPG users, there is evidence of potential short-term respiratory health benefits when transitioning from partial to exclusive use of cleaner cooking fuels. For most symptoms, the increased odds among women stacking LPG and polluting fuels were similar in magnitude to the odds of symptoms among those exclusively using polluting fuels. This indicates a minimal health benefit to the cook when partially displacing their use of polluting fuels with LPG.

The strongest association between cooking fuel type and respiratory symptoms occurred in Eldoret, Kenya. Eldoret was the only community where the lowest prevalence of each respiratory outcome occurred among exclusive LPG users (Fig. 3). This finding is consistent with results from household air pollution (HAP) monitoring that occurred among the same study population; HAP measurements showed that cooking contributed more to overall PM_2.5_ cook exposures in Eldoret compared with Obuasi and Cameroon [46].AP exposure contrasts between LPG primary and polluting fuel exclusive users were also highest in Eldoret (Table 2), further supporting the notion that higher PM_2.5_ levels among polluting fuel users is potentially driving the higher prevalence of respiratory symptoms.

The lack of a clear trend in the prevalence of respiratory symptoms by primary and secondary cooking fuel type in Obuasi and Mbalmayo (Fig. 3) may be due to other PM_2.5_ sources contributing to a larger proportion of overall exposures in those two communities [46].

## 5 Confounding by secondary cooking fuel type

We found that secondary cooking fuel type confounded the association between primary cooking fuel type and headache; there was not a significant association between primary cooking fuel type and odds of headaches (OR 1.01 95 % CI 0.87 – 1.18) (Supplementary Table 15), yet a significantly higher odds of headache was detected among women stacking LPG and polluting fuels (OR 1.35, 95 % CI 1.01–1.80) compared with exclusive LPG users. In addition, we found that using LPG for cooking was significantly associated with reduced eye irritation symptoms when used 100 % of the time [OR 0.43, 95 % CI 0.26–0.72]. The negative association remained but was not statistically significant among households using LPG for only some of their cooking (Supplementary Table 21). These results further suggest that partial displacement of polluting cooking fuels with LPG use is insufficient to alleviate several short-term health effects associated with exposure to biomass smoke. Thus, future survey-based studies should aim to collect information on all (e.g., primary and secondary) cooking fuels used to better assess the impact of cooking on respiratory health.

## 6 High prevalence of fuel stacking

While our study builds upon the finding of other research that a complete transition to clean fuels is needed to maximize the health benefits, [47] it is important to acknowledge that complete transitions to clean fuels are rare in LMICs, with the prevalence of stacking typically ranging from 30 to 100 % [26,28]. Thus, governments should also consider programs that can promote "cleaner stacking" through use of multiple appliances that respond to locally relevant household needs. Additionally, given the proportion of fuel switching documented in this study, policies are needed to mitigate interruptions to clean fuel affordability, due to seasonal [48] or external price increases [49].

## 7 Short-term vs long-term health symptoms

Given that the symptoms included in this study are reflect short-term adverse health effects, highlighting the drastically lower prevalence of certain conditions (e.g., shortness of breath) may be useful for promoting the use of LPG for cooking. Increasing awareness about an immediate reduction in burdensome symptoms may increase women's willingness to adopt LPG, compared with knowledge of a reduction in long-term CVD or respiratory health risks that are not immediately realised. Additionally, the respiratory symptoms reported in this study (e.g. coughing, wheezing) may indicate a higher likelihood of future serious pulmonary illness such as COPD [50,51].

### 7.1 Headaches

The high (65 %) prevalence of headaches in the study population (Supplementary Table 2) has been reported in other African countries [52] and among cooks in Latin America [53] and rural India. [54] In another study conducted in rural Kenya, the presence of headaches was nearly universal (98 %) [55].

We further uncovered that the rate of headaches was 30 % lower among individuals exclusively using LPG compared with those stacking fuels; other cross-sectional studies have noted significantly higher prevalence of headache among polluting fuel users compared with clean fuel users [56]. A longitudinal study in Guatemala additionally found that 88 % of women reported a reduction in headache when switching away from traditional wood stoves for cooking [57]. A consistently higher rate of headaches among polluting fuel users relative to exclusive LPG users may be due to higher carbon monoxide exposure from biomass burning [14] or the physical burden of carrying firewood for long distances [54].

We note that higher disparities in headache prevalence between exclusive LPG and exclusive polluting fuel users in the Kenyan and Cameroonian communities compared with the Ghanian setting may be due to a greater contribution of HAP to overall PM_2.5_ cook exposures in Mbalmayo and Eldoret [46].

We also detected a higher prevalence of headache among primary cooks stacking LPG with polluting fuels (82 %) compared with exclusive polluting fuel users (72 %) in Mbalmayo. This finding may be due to confounding, as there may be a wider range of risk factors for headaches, aside from HAP (e.g. stress-induced, hot climate, hormone fluctuations, sleep deprivation), relative to that of respiratory symptoms [58].

Headaches can have a substantial negative impact on an individual's daily activities; a study in Ethiopia found that 68 % present of participants suffering from headaches reported missing work, school or social events [59]. Numerous studies have also reported that women are nearly three times as likely to experience headaches than men [60]. A 30 % lower odds of headache among women exclusively cooking with LPG compared with those stacking LPG and polluting fuels in Mbalmayo and Eldoret suggests that facilitating a complete transition to clean cooking fuels may reduce the gender gap in headache prevalence.

### 7.2 Other factors impacting health symptoms

Although some health symptoms (e.g. chest tightness, wheezing and cough) were not higher among women cooking with polluting fuels, the symptoms were more pronounced among women without access to electricity for lighting. There was also a significantly lower prevalence of several health symptoms (eye irritation, headaches, shortness of breath and wheezing) among women exclusively using polluting fuels in Mbalmayo that used electricity for lighting compared to those without access (Fig. 4). This suggests that electrification can also be an effective intervention for improving short-term respiratory health, even in households where clean cooking fuels are not used.

Previous studies conducted in sub-Saharan Africa have found that households using clean fuels for lighting (solar-powered lamps) as opposed to kerosene lamps had significantly lower levels of HAP in their home and that the risk of cough and illness was reduced [61].

We also found that exposure to second-hand smoke was associated with increased odds of several respiratory health symptoms. In another multinational HAP study conducted among peri‑urban communities, second-hand smoke was found to be a significant predictor of PM_2.5_ exposures among women [62], reinforcing its substantial contribution to air pollution exposures, even in households where biomass smoke is present. There is therefore a need to target multiple sources of indoor air pollution to alleviate respiratory symptoms among the primary cook of the household.

### 7.3 Strengths and limitations

This multinational study is one of the first to investigate the impacts of cooking fuel stacking on health symptoms of female cooks. While the outcomes are subject to self-reporting bias, the use of survey data represents a useful proxy for assessing the potential impact of polluting fuel use on respiratory disease in low-income areas [63] where objective measures of respiratory health are costly and difficult to collect. Given the large sample size, this study was sufficiently powered to detect differences in several outcomes by primary and secondary cooking fuel type.

Since this study was cross-sectional, a causal relationship between fuel stacking and adverse health outcomes cannot be ascertained. This study also did not collect information on all relevant potential confounding variables that can affect headaches or respiratory health, such as physical activity levels, occupational air pollution exposure and proximity to other sources of outdoor pollution, including trash burning; therefore, there is the potential for residual confounding. However, we note that a randomized trial likewise detected substantial reductions in health symptoms following displacement of traditional wood stoves for cooking [14]. As this study was limited to female main cooks in peri‑urban communities, the effect of fuel stacking on the health status of males and individuals in rural communities may be different.

Although LPG was used exclusively by only 10 % of the study population (*n* = 118), the large sample size of more than 1100 individuals provided statistical power for comparing the prevalence of headaches, eye irritation and respiratory symptoms among exclusive LPG users, exclusive polluting fuel users and those stacking clean and polluting cooking fuels.

## 8 Conclusions

The marginal reduction in the prevalence of health symptoms among those stacking LPG and polluting fuels relative to exclusive polluting fuel users suggests that there is some health benefit to ‘cleaner stacking’. However, to further reduce the prevalence of burdensome health symptoms among primary cooks, policymakers should ultimately aim to eliminate the use of polluting cooking fuels in traditional stoves. Increasing access to LPG as a cleaner option for cooking in sub-Saharan Africa will require substantial investment that can be directed toward lowering LPG refill costs [[64], [65], [66]], increasing the density of LPG retailers [40,67] and expanding flexible payment options, such as pay-as-you-go LPG [68].

We additionally uncovered large between-community discrepancies in the difference in headache, eye irritation and respiratory symptom prevalence between primary LPG and exclusive polluting fuel users. It is possible that community-level differences in the relative contribution of emissions from cooking to overall PM_2.5_ exposures [46] in the study settings may be responsible for this variation in respiratory symptom prevalence. Additional studies that conduct of HAP monitoring alongside the collection of information on respiratory outcomes across different fuel types are needed in sub-Saharan Africa to improve understanding of determinants of disparities in health symptom prevalence.

Beyond minimizing exposure to biomass burning, the significant association between a lack of access to an electricity connection for lighting and exposure to second-hand smoke and several respiratory symptoms identifies other sources of indoor air pollution that should be targeted for mitigation to potentially also help improve respiratory health among women in peri‑urban sub-Saharan Africa.

## Financial disclosure statement

No relevant financial relationship exists.

## CRediT authorship contribution statement

**Kourosh Parvizi:** Formal analysis, Investigation, Writing – original draft. **Diana Menya:** Project administration, Resources. **Emily Nix:** Data curation, Writing – review & editing. **Judith Mangeni:** Project administration. **Federico Lorenzetti:** Data curation. **Edna Sang:** Project administration. **Rachel Anderson de Cuevas:** Project administration. **Theresa Tawiah:** Project administration, Resources. **Miranda Baame:** Project administration, Resources. **Emmanuel Betang:** Project administration, Resources. **Sara Ronzi:** Project administration. **Mieks Twumasi:** Project administration. **Seeba Amenga-Etego:** Project administration. **Reginald Quansah:** Project administration. **Bertrand Hugo Mbatchou Ngahane:** Project administration. **Elisa Puzzolo:** Funding acquisition, Project administration, Writing – review & editing. **Kwaku Poku Asante:** Project administration. **Daniel Pope:** Funding acquisition, Writing – review & editing. **Matthew Shupler:** Conceptualization, Data curation, Investigation, Methodology, Project administration, Supervision, Writing – review & editing.

## Declaration of competing interest

The authors have no conflicts of interest to declare.

## Data Availability

Data will be made available on request.

## Appendix Supplementary materials

Image, application 1
