# Supplementary material for: Burden of headaches, eye irritation and respiratory symptoms among females stacking LPG with polluting cooking fuels: Modelling from peri-urban Cameroon, Ghana & Kenya
Source: Energy Nexus. 2024 Jul;14:None. doi: 10.1016/j.nexus.2024.100304 (PMC11177547; doi:10.1016/j.nexus.2024.100304)
Supplement: Supplementary file 1 [file mmc1.docx]

**Supplemental Information**

Burden of headaches, eye irritation and respiratory symptoms among females stacking LPG with polluting cooking fuels: Modelling from peri-urban Cameroon, Ghana & Kenya

Kourosh Parvizi^1^, Diana Menya^2^, Emily Nix^1^, Judith Mangeni^2^, Federico Lorenzetti^1^, Edna Sang^2^, Rachel Anderson de Cuevas^1^, Theresa Tawiah^3^, Miranda Baame^4^, Emmanuel Betang^4^, Sara Ronzi^1^, Mieks Twumasi^3^, Seeba Amenga-Etego^3^, Reginald Quansah^5^, Bertrand Hugo Mbatchou Ngahane^4^, Elisa Puzzolo^1^, Kwaku Poku Asante^3^, Daniel Pope^1^, Matthew Shupler^1*^

^1^Department of Public Health, Policy and Systems, University of Liverpool, United Kingdom

^2^School of Public Health, Moi University, Eldoret, Kenya

^3^Kintampo Health Research Centre, Kintampo, Ghana

^4^Douala General Hospital, Douala, Cameroon

^5^School of Public Health, University of Ghana, Ghana

*Corresponding author: [m.shupler@liverpool.ac.uk](mailto:m.shupler@liverpool.ac.uk)

**Supplementary Table 1 –** In-depth survey questions used to assess symptoms and presence of chronic disease

| **Question** | **Outcome assessed** |
| --- | --- |
| Have you, at any time in the last 12 months, had wheezing or whistling in your chest? | Wheezing |
| Have you, at any time in the last 12 months, woken up with a feeling of tightness in your chest first thing in the morning? | Tightness |
| Have you, at any time in the last 12 months, had an attack of shortness of breath that came on during the day when you were not doing anything strenuous? | Shortness of breath |
| Have you, at any time in the last 12 months, had an attack of shortness of breath that came on after you had stopped doing something strenuous? |  |
| Have you, at any time in the last 12 months, been woken at night by an attack of shortness of breath? |  |
| Have you, at any time in the last 12 months, been woken at night by an attack of coughing? | Cough |
| Do you usually cough first thing in the morning? |  |
| Do you have a cough like this most mornings for as much as 3 months per year? |  |
| Do you usually cough up phlegm from your chest first thing in the morning? |  |
| Do you have phlegm like this most mornings for as much as 3 months per year? |  |
| Over the last 12 months, have you experienced headaches? | Headaches |
| Over the last 12 months, have you experienced eye irritation when cooking? | Eye irritation |
| Have you ever been told by a doctor that you have Tuberculosis? | Chronic disease |
| Have you ever been told by a doctor that you have Chronic bronchitis? |  |
| Have you ever been told by a doctor that you have heart disease? |  |
| Have your ever been told by a doctor or other health worker that you have raised blood pressure or hypertension? |  |

**Supplementary table 2** – Prevalence of recorded symptoms amongst study population

|  | Total  (n = 1147) | Mbalmyo  (n = 403) | Obuasi  (n = 347) | Eldoret  (n = 397) |
| --- | --- | --- | --- | --- |
| **Symptom, n (%)** |  |  |  |  |
| Wheezing | 149 (13.0) | 73 (18.1) | 25 (14.7) | 51 (12.8) |
| Tightness | 145 (12.6) | 85 (21.1) | 11 (14.1) | 49 (12.3) |
| Shortness of breath | 165 (14.4) | 61 (15.1) | 49 (15.9) | 55 (13.9) |
| Cough | 194 (16.9) | 68 (16.9) | 54 (20.7) | 72 (18.1) |
| Eye irritation | 378 (33.0) | 240 (59.6) | 49 (25.6) | 89 (22.4) |
| Headaches | 745 (65.0) | 308 (76.4) | 196 (69.5) | 241 (60.7) |

**Supplementary Table 3 -** Model select based on optimising the Akaike Information Criterion (AIC) and coefficient of determination (R^2^)

| Model | Variables included | Conditional R2 | Marginal R2 | Change in Marginal R2 | ICC | AIC |
| --- | --- | --- | --- | --- | --- | --- |
| 1 | Base random effects | 0.183 | 0 | / | 0.183 | 1487.451 |
| 2 | Base + primary fuel type | 0.203 | 0.028 | 0.028 | 0.18 | 1469.809 |
| 3 | Base + primary fuel type + demographic variables | 0.204 | 0.041 | 0.013 | 0.17 | 1466.479 |
| 4 | Base + primary fuel type + demographic variables + financial security | 0.196 | 0.054 | 0.013 | 0.15 | 1465.254 |
| 5 | Base + primary fuel type + demographic variables + financial security + energy poverty | 0.194 | 0.069 | 0.015 | 0.134 | 1459.435 |
| **6** | **Base + primary fuel type + demographic variables + financial security + energy poverty + chronic condition + other household smokers** | **0.197** | **0.072** | **0.003** | **0.133** | **1452.737** |


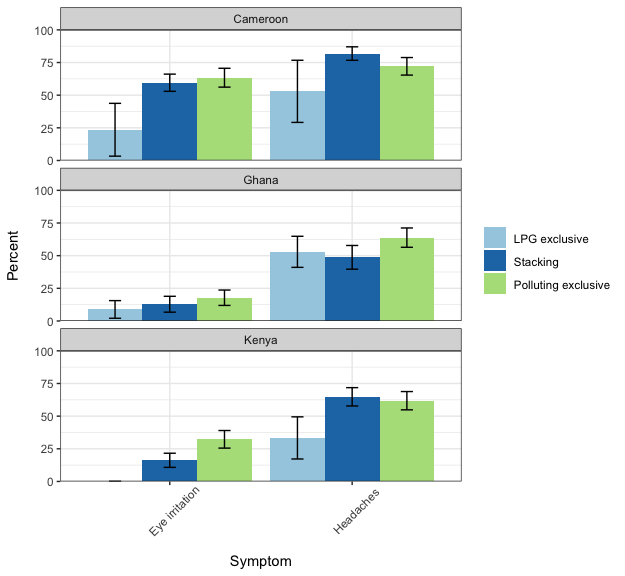


**Supplementary figure 1 -** Prevalence of non-respiratory symptoms in whole study population, stratified by community and stacking behaviour.

**Supplementary Table 4 -** Effect of fuel choice on prevalence of wheezing (Primary fuel model). Data is presented as odds ratio (OR) with 95% confidence intervals (95% CI) obtained from a multivariate Poisson logistic regression adjusting for confounding variables. Columns from left to right; Predictor (see Supplementary Table 3), OR of predictor, 95% CI of OR, p value.

| \|  \| **Odds of wheezing** \| \| \| \| --- \| --- \| --- \| --- \| \| *Predictors* \| *Odds ratio* \| *95% CI* \| *p* \| \| (Intercept) \| 0.10 \| 0.03 – 0.33 \| **<0.001** \| \| Polluting fuel \| 1.11 \| 0.79 – 1.56 \| 0.557 \| \| Age \| 1.00 \| 0.98 – 1.01 \| 0.816 \| \| Education level: Primary school \| 1.36 \| 0.52 – 3.56 \| 0.528 \| \| Education level: Secondary school \| 1.45 \| 0.56 – 3.74 \| 0.447 \| \| Education level: University \| 0.80 \| 0.27 – 2.37 \| 0.684 \| \| Financial security: Definitely not financially secure \| 1.41 \| 0.81 – 2.45 \| 0.227 \| \| Financial security: Not quite financially secure \| 1.44 \| 0.87 – 2.39 \| 0.161 \| \| Other smokers in the household \| 1.76 \| 1.06 – 2.91 \| **0.029** \| \| Chronic health condition* \| 1.40 \| 0.91 – 2.18 \| 0.130 \| \| No electricity connection \| 1.87 \| 1.29 – 2.71 \| **0.001** \| |  |
| --- | --- | --- | --- | --- | --- | --- | --- | --- | --- | --- | --- | --- | --- | --- | --- | --- | --- | --- | --- | --- | --- | --- | --- | --- | --- | --- | --- | --- | --- | --- | --- | --- | --- | --- | --- | --- | --- | --- | --- | --- | --- | --- | --- | --- | --- | --- | --- | --- | --- | --- | --- | --- | --- |

**Supplementary Table 5 -** Effect of fuel choice on prevalence of wheezing (Stacking model). Data is presented as odds ratio (OR) with 95% confidence intervals (95% CI) obtained from a multivariate Poisson logistic regression adjusting for confounding variables. Columns from left to right; Predictor (see Supplementary Table 3), OR of predictor, 95% CI of OR, p value.

|  | **Odds of wheezing** | | |
| --- | --- | --- | --- |
| *Predictors* | *Odds ratio* | *CI* | *p* |
| (Intercept) | 0.11 | 0.03 – 0.41 | **0.001** |
| Using polluting fuels exclusively | 0.97 | 0.51 – 1.83 | 0.915 |
| Stacking | 0.98 | 0.51 – 1.88 | 0.948 |
| Age | 1.00 | 0.98 – 1.01 | 0.879 |
| Education level: Primary school | 1.35 | 0.52 – 3.53 | 0.541 |
| Education level: Secondary school | 1.41 | 0.55 – 3.67 | 0.475 |
| Education level: University | 0.76 | 0.25 – 2.28 | 0.626 |
| Financial security: Definitely not financially secure | 1.44 | 0.83 – 2.50 | 0.200 |
| Financial security: Not quite financially secure | 1.46 | 0.88 – 2.43 | 0.141 |
| Other smokers in the household | 1.77 | 1.07 – 2.94 | **0.026** |
| Chronic health condition* | 1.41 | 0.91 – 2.19 | 0.125 |
| No electricity connection | 1.88 | 1.29 – 2.72 | **0.001** |


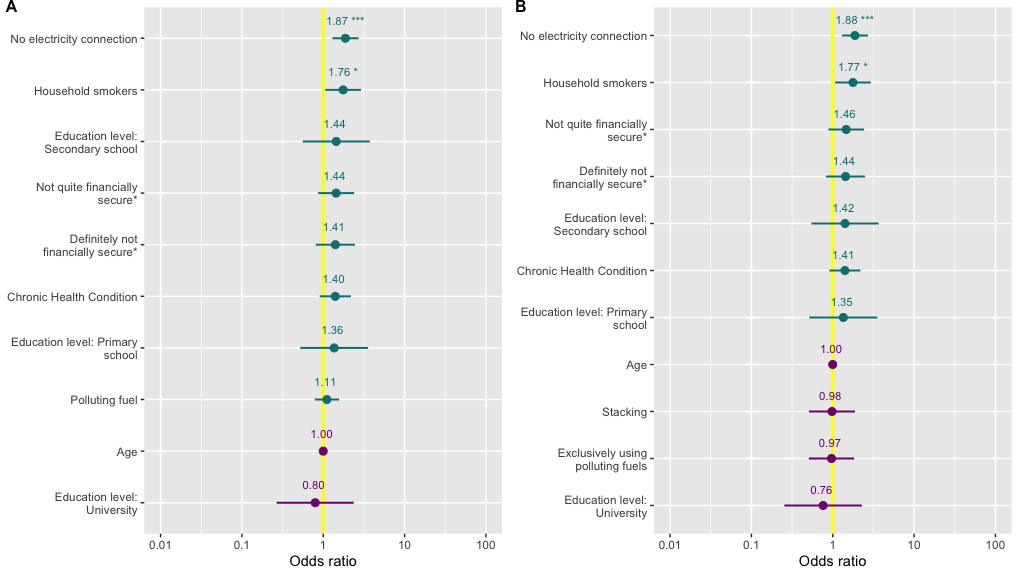


**Supplementary Figure 2.**  Odds (95% CI) of wheezing for primary fuel model (A) and stacking model (B) * Not quite financially secure represents individuals reporting “Not quite enough money” to live. ** Definitely not financially secure represents individuals reporting “Definitely not enough money” to live.

**Supplementary Table 6 -** Effect of fuel choice on prevalence of tightness (Primary fuel model). Data is presented as odds ratio (OR) with 95% confidence intervals (95% CI) obtained from a multivariate Poisson logistic regression adjusting for confounding variables. Columns from left to right; Predictor (see Supplementary Table 3), OR of predictor, 95% CI of OR, p value.

|  | **Odds of having chest tightness** | | |
| --- | --- | --- | --- |
| *Predictors* | *Odds ratio* | *CI* | *p* |
| (Intercept) | 0.14 | 0.03 – 0.58 | **0.007** |
| Polluting fuel | 0.99 | 0.70 – 1.40 | 0.962 |
| Age | 0.99 | 0.98 – 1.01 | 0.337 |
| Education level: Primary school | 1.03 | 0.36 – 2.90 | 0.962 |
| Education level: Secondary school | 1.20 | 0.43 – 3.36 | 0.723 |
| Education level: University | 0.44 | 0.14 – 1.43 | 0.174 |
| Financial security: Definitely not financially secure | 0.85 | 0.49 – 1.49 | 0.571 |
| Financial security: Not quite financially secure | 1.18 | 0.73 – 1.92 | 0.494 |
| Other smokers in the household | 1.92 | 1.19 – 3.11 | **0.008** |
| Chronic health condition* | 2.01 | 1.35 – 3.01 | **0.001** |
| No electricity connection | 1.69 | 1.20 – 2.37 | **0.003** |

**Supplementary Table 7 -** Effect of fuel choice on prevalence of tightness (Stacking model). Data is presented as odds ratio (OR) with 95% confidence intervals (95% CI) obtained from a multivariate Poisson logistic regression adjusting for confounding variables. Columns from left to right; Predictor (see Supplementary Table 3), OR of predictor, 95% CI of OR, p value.

|  | **Odds of having chest tightness** | | |
| --- | --- | --- | --- |
| *Predictors* | *Odds ratio* | *CI* | *p* |
| (Intercept) | 0.11 | 0.02 – 0.54 | **0.007** |
| Using polluting fuels exclusively | 1.21 | 0.55 – 2.66 | 0.640 |
| Stacking | 1.33 | 0.61 – 2.93 | 0.473 |
| Age | 0.99 | 0.98 – 1.01 | 0.346 |
| Education level: Primary school | 1.03 | 0.36 – 2.90 | 0.962 |
| Education level: Secondary school | 1.20 | 0.43 – 3.35 | 0.729 |
| Education level: University | 0.44 | 0.13 – 1.42 | 0.168 |
| Financial security: Definitely not financially secure | 0.86 | 0.49 – 1.50 | 0.595 |
| Financial security: Not quite financially secure | 1.20 | 0.74 – 1.94 | 0.462 |
| Other smokers in the household | 1.92 | 1.19 – 3.11 | **0.008** |
| Chronic health condition* | 2.01 | 1.34 – 3.01 | **0.001** |
| No electricity connection | 1.68 | 1.19 – 2.35 | **0.003** |


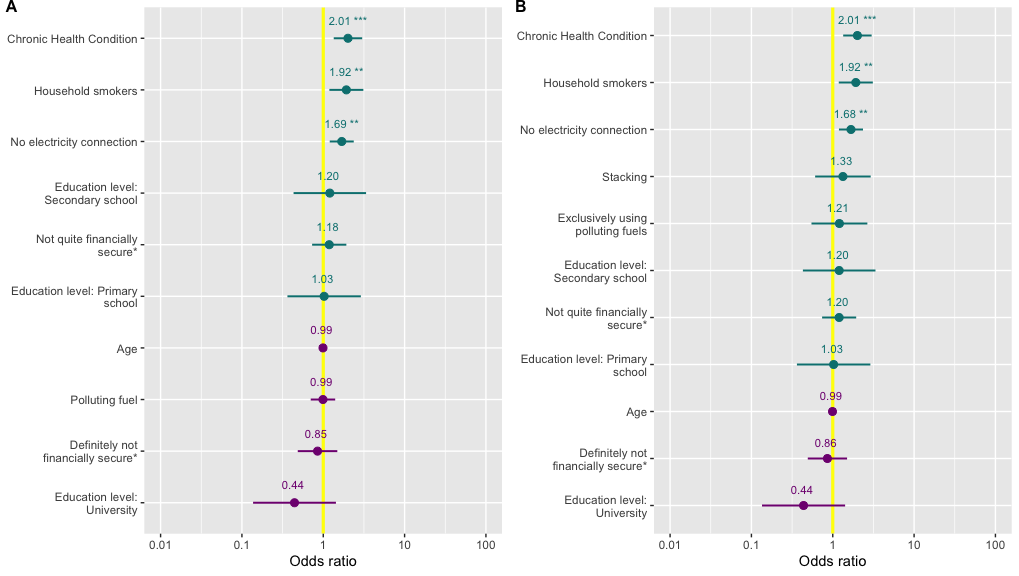


**Supplementary Figure 3**. Odds (95% CI) of chest tightness for primary fuel model (A) and stacking model (B) * Not quite financially secure represents individuals reporting “Not quite enough money” to live. ** Definitely not financially secure represents individuals reporting “Definitely not enough money” to live.

**Supplementary Table 8 -** Effect of fuel choice on prevalence of shortness of breath (Primary fuel model). Data is presented as odds ratio (OR) with 95% confidence intervals (95% CI) obtained from a multivariate Poisson logistic regression adjusting for confounding variables. Columns from left to right; Predictor (see Supplementary Table 3), OR of predictor, 95% CI of OR, p value.

|  | **Odds of having shortness of breath** | | |
| --- | --- | --- | --- |
| *Predictors* | *Odds ratio* | *CI* | *p* |
| (Intercept) | 0.15 | 0.06 – 0.40 | **<0.001** |
| Polluting fuel | 0.83 | 0.60 – 1.14 | 0.253 |
| Age | 1.00 | 0.99 – 1.02 | 0.702 |
| Education level: Primary school | 0.82 | 0.39 – 1.70 | 0.588 |
| Education level: Secondary school | 0.96 | 0.48 – 1.94 | 0.911 |
| Education level: University | 0.67 | 0.30 – 1.50 | 0.331 |
| Financial security: Definitely not financially secure | 0.83 | 0.48 – 1.42 | 0.498 |
| Financial security: Not quite financially secure | 1.72 | 1.12 – 2.64 | **0.014** |
| Other smokers in the household | 1.45 | 0.84 – 2.48 | 0.179 |
| Chronic health condition* | 2.05 | 1.41 – 2.98 | **<0.001** |
| No electricity connection | 1.69 | 1.23 – 2.33 | **0.001** |

**Supplementary Table 9 -** Effect of fuel choice on prevalence of shortness of breath (Stacking model). Data is presented as odds ratio (OR) with 95% confidence intervals (95% CI) obtained from a multivariate Poisson logistic regression adjusting for confounding variables. Columns from left to right; Predictor (see Supplementary Table 3), OR of predictor, 95% CI of OR, p value.

|  | **Odds of having shortness of breath** | | |
| --- | --- | --- | --- |
| *Predictors* | *Odds ratio* | *CI* | *p* |
| (Intercept) | 0.08 | 0.02 – 0.27 | **<0.001** |
| Using polluting fuels exclusively | 1.57 | 0.75 – 3.30 | 0.233 |
| Stacking | 2.16 | 1.04 – 4.48 | **0.038** |
| Age | 1.00 | 0.99 – 1.02 | 0.785 |
| Education level: Primary school | 0.78 | 0.38 – 1.62 | 0.506 |
| Education level: Secondary school | 0.91 | 0.45 – 1.84 | 0.792 |
| Education level: University | 0.63 | 0.28 – 1.43 | 0.271 |
| Financial security: Definitely not financially secure | 0.81 | 0.47 – 1.39 | 0.441 |
| Financial security: Not quite financially secure | 1.69 | 1.10 – 2.59 | **0.016** |
| Other smokers in the household | 1.44 | 0.84 – 2.47 | 0.187 |
| Chronic health condition* | 2.03 | 1.40 – 2.96 | **<0.001** |
| No electricity connection | 1.61 | 1.17 – 2.22 | **0.004** |


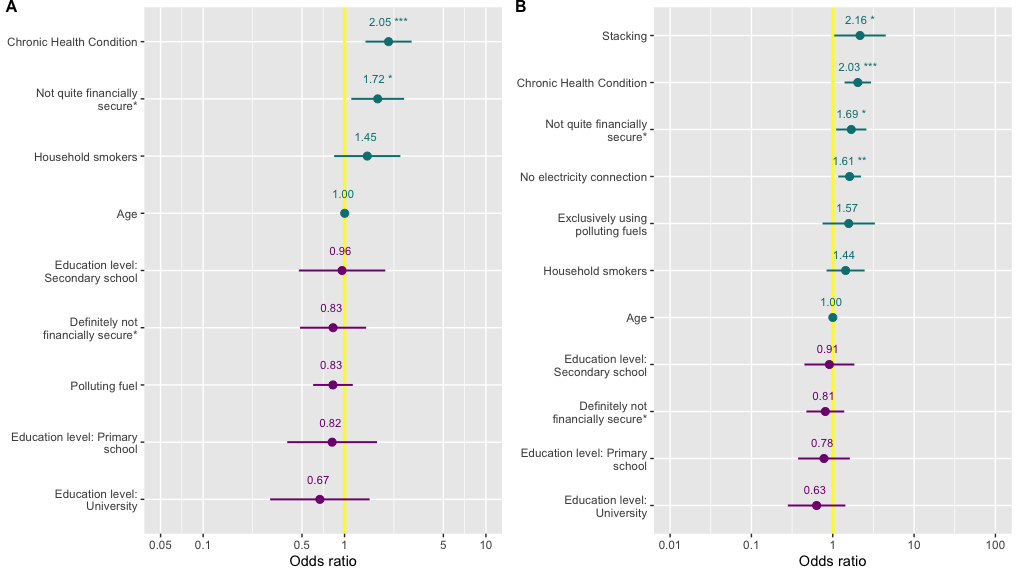


**Supplementary Figure 4**. Odds (95% CI) of shortness of breath for primary fuel model (A) and stacking model (B) * Not quite financially secure represents individuals reporting “Not quite enough money” to live. ** Definitely not financially secure represents individuals reporting “Definitely not enough money” to live.

**Supplementary Table 10 -** Effect of fuel choice on prevalence of cough (Primary fuel model). Data is presented as odds ratio (OR) with 95% confidence intervals (95% CI) obtained from a multivariate Poisson logistic regression adjusting for confounding variables. Columns from left to right; Predictor (see Supplementary Table 3), OR of predictor, 95% CI of OR, p value.

|  | **Odds of coughing** | | |
| --- | --- | --- | --- |
| *Predictors* | *Odds ratio* | *CI* | *p* |
| (Intercept) | 0.19 | 0.08 – 0.47 | **<0.001** |
| Polluting fuel | 1.31 | 0.97 – 1.78 | 0.083 |
| Age | 0.99 | 0.98 – 1.01 | 0.282 |
| Education level: Primary school | 0.91 | 0.47 – 1.75 | 0.778 |
| Education level: Secondary school | 0.87 | 0.46 – 1.65 | 0.673 |
| Education level: University | 0.55 | 0.26 – 1.18 | 0.123 |
| Financial security: Definitely not financially secure | 0.90 | 0.56 – 1.45 | 0.662 |
| Financial security: Not quite financially secure | 1.56 | 1.05 – 2.32 | **0.028** |
| Other smokers in the household | 1.78 | 1.13 – 2.80 | **0.012** |
| Chronic health condition* | 1.79 | 1.25 – 2.57 | **0.002** |
| No electricity connection | 1.40 | 1.04 – 1.89 | **0.027** |

**Supplementary Table 11 -** Effect of fuel choice on prevalence of cough (Stacking model). Data is presented as odds ratio (OR) with 95% confidence intervals (95% CI) obtained from a multivariate Poisson logistic regression adjusting for confounding variables. Columns from left to right; Predictor (see Supplementary Table 3), OR of predictor, 95% CI of OR, p value.

|  | **Odds of coughing** | | |
| --- | --- | --- | --- |
| *Predictors* | *Odds ratio* | *CI* | *p* |
| (Intercept) | 0.15 | 0.05 – 0.42 | **<0.001** |
| Using polluting fuels exclusively | 1.65 | 0.90 – 3.02 | 0.106 |
| Stacking | 1.29 | 0.70 – 2.39 | 0.413 |
| Age | 0.99 | 0.98 – 1.01 | 0.325 |
| Education level: Primary school | 0.93 | 0.49 – 1.80 | 0.840 |
| Education level: Secondary school | 0.90 | 0.47 – 1.71 | 0.745 |
| Education level: University | 0.57 | 0.26 – 1.22 | 0.149 |
| Financial security: Definitely not financially secure | 0.91 | 0.57 – 1.47 | 0.706 |
| Financial security: Not quite financially secure | 1.56 | 1.05 – 2.32 | **0.027** |
| Other smokers in the household | 1.76 | 1.12 – 2.77 | **0.014** |
| Chronic health condition* | 1.78 | 1.24 – 2.56 | **0.002** |
| No electricity connection | 1.39 | 1.03 – 1.88 | **0.031** |


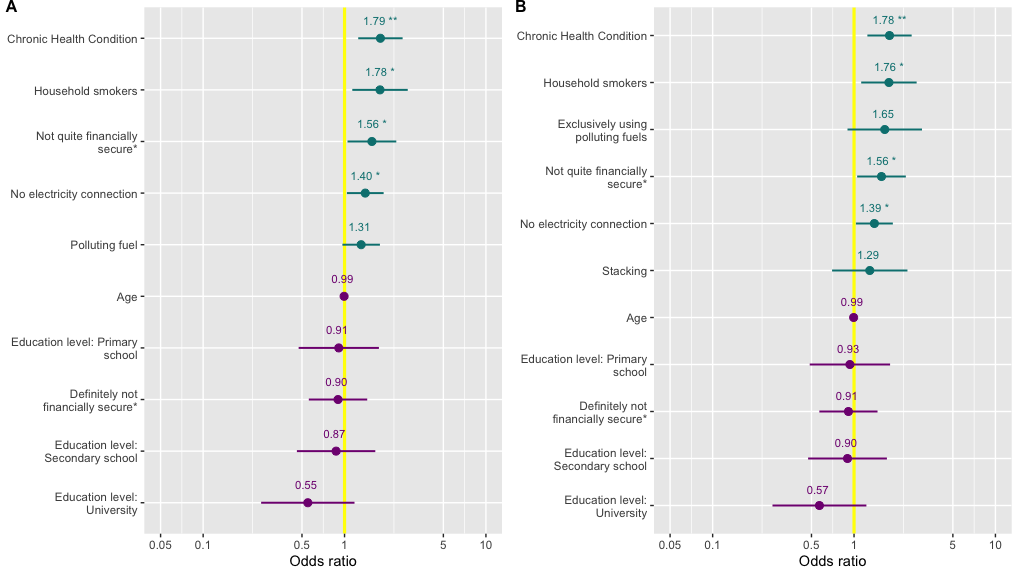


**Supplementary Figure 5**  - Odds (95% CI) of coughing for primary fuel model (A) and stacking model (B) * Not quite financially secure represents individuals reporting “Not quite enough money” to live. ** Definitely not financially secure represents individuals reporting “Definitely not enough money” to live.

**Supplementary Table 12 -** Effect of fuel choice on prevalence of eye irritation (Primary fuel model). Data is presented as odds ratio (OR) with 95% confidence intervals (95% CI) obtained from a multivariate Poisson logistic regression adjusting for confounding variables. Columns from left to right; Predictor (see Supplementary Table 3), OR of predictor, 95% CI of OR, p value.

|  | **Odds of having eye irritation** | | |
| --- | --- | --- | --- |
| *Predictors* | *Odds ratio* | *CI* | *p* |
| (Intercept) | 0.10 | 0.04 – 0.28 | **<0.001** |
| Polluting fuel | 1.44 | 1.15 – 1.80 | **0.001** |
| Age | 1.01 | 1.00 – 1.02 | 0.107 |
| Education level: Primary school | 1.59 | 0.82 – 3.07 | 0.170 |
| Education level: Secondary school | 1.40 | 0.72 – 2.70 | 0.322 |
| Education level: University | 1.25 | 0.61 – 2.58 | 0.546 |
| Financial security: Definitely not financially secure | 1.48 | 1.02 – 2.14 | **0.039** |
| Financial security: Not quite financially secure | 1.38 | 0.98 – 1.96 | 0.067 |
| Other smokers in the household | 1.18 | 0.84 – 1.67 | 0.347 |
| Chronic health condition* | 1.24 | 0.95 – 1.63 | 0.117 |
| No electricity connection | 1.35 | 1.09 – 1.67 | **0.006** |

**Supplementary Table 13 -** Effect of fuel choice on prevalence of eye irritation (Stacking model). Data is presented as odds ratio (OR) with 95% confidence intervals (95% CI) obtained from a multivariate Poisson logistic regression adjusting for confounding variables. Columns from left to right; Predictor (see Supplementary Table 3), OR of predictor, 95% CI of OR, p value.

|  | **Odds of having eye irritation** | | |
| --- | --- | --- | --- |
| *Predictors* | *Odds ratio* | *CI* | *p* |
| (Intercept) | 0.05 | 0.01 – 0.15 | **<0.001** |
| Using polluting fuels exclusively | 2.87 | 1.51 – 5.45 | **0.001** |
| Stacking | 2.45 | 1.29 – 4.67 | **0.006** |
| Age | 1.01 | 1.00 – 1.02 | 0.065 |
| Education level: Primary school | 1.65 | 0.85 – 3.19 | 0.137 |
| Education level: Secondary school | 1.45 | 0.75 – 2.80 | 0.274 |
| Education level: University | 1.25 | 0.61 – 2.59 | 0.544 |
| Financial security: Definitely not financially secure | 1.55 | 1.07 – 2.23 | **0.020** |
| Financial security: Not quite financially secure | 1.43 | 1.01 – 2.02 | **0.042** |
| Other smokers in the household | 1.17 | 0.83 – 1.65 | 0.372 |
| Chronic health condition* | 1.25 | 0.95 – 1.64 | 0.105 |
| No electricity connection | 1.32 | 1.06 – 1.63 | **0.012** |

**Supplementary Table 14 -** Effect of fuel choice on prevalence of headaches (Primary fuel model). Data is presented as odds ratio (OR) with 95% confidence intervals (95% CI) obtained from a multivariate Poisson logistic regression adjusting for confounding variables. Columns from left to right; Predictor (see Supplementary Table 3), OR of predictor, 95% CI of OR, p value.

|  | **Odds of having headaches** | | |
| --- | --- | --- | --- |
| *Predictors* | *Odds ratio* | *CI* | *p* |
| (Intercept) | 0.47 | 0.28 – 0.78 | **0.004** |
| Polluting fuel | 1.01 | 0.87 – 1.18 | 0.871 |
| Age | 1.00 | 0.99 – 1.01 | 0.612 |
| Education level: Primary school | 1.44 | 0.96 – 2.17 | 0.078 |
| Education level: Secondary school | 1.30 | 0.87 – 1.95 | 0.203 |
| Education level: University | 1.25 | 0.80 – 1.94 | 0.321 |
| Financial security: Definitely not financially secure | 1.01 | 0.81 – 1.27 | 0.916 |
| Financial security: Not quite financially secure | 1.08 | 0.89 – 1.31 | 0.442 |
| Other smokers in the household | 0.95 | 0.71 – 1.29 | 0.763 |
| Chronic health condition* | 1.20 | 0.97 – 1.47 | 0.087 |
| No electricity connection | 1.15 | 0.97 – 1.35 | 0.105 |

**Supplementary Table 15 -** Effect of fuel choice on prevalence of headaches (Stacking model). Data is presented as odds ratio (OR) with 95% confidence intervals (95% CI) obtained from a multivariate Poisson logistic regression adjusting for confounding variables. Columns from left to right; Predictor (see Supplementary Table 3), OR of predictor, 95% CI of OR, p value.

|  | **Odds of having headaches** | | |
| --- | --- | --- | --- |
| *Predictors* | *Odds ratio* | *CI* | *p* |
| (Intercept) | 0.36 | 0.21 – 0.64 | **<0.001** |
| Using polluting fuels exclusively | 1.31 | 0.98 – 1.75 | 0.068 |
| Stacking | 1.35 | 1.01 – 1.80 | **0.041** |
| Age | 1.00 | 0.99 – 1.00 | 0.575 |
| Education level: Primary school | 1.45 | 0.97 – 2.19 | 0.071 |
| Education level: Secondary school | 1.32 | 0.88 – 1.98 | 0.183 |
| Education level: University | 1.25 | 0.81 – 1.95 | 0.313 |
| Financial security: Definitely not financially secure | 1.02 | 0.81 – 1.28 | 0.856 |
| Financial security: Not quite financially secure | 1.08 | 0.89 – 1.31 | 0.428 |
| Other smokers in the household | 0.95 | 0.71 – 1.29 | 0.765 |
| Chronic health condition* | 1.19 | 0.97 – 1.47 | 0.092 |
| No electricity connection | 1.13 | 0.96 – 1.34 | 0.132 |

**Supplementary Table 16 -** Effect of fuel use behaviour on prevalence of coughing, comparing self-reported time spent cooking using LPG as a percentage of total cooking time (reference = 0% time spent using LPG). Data is presented as odds ratio (OR) with 95% confidence intervals (95% CI) obtained from a multivariate Poisson logistic regression adjusting for confounding variables. Columns from left to right; Predictor (see Supplementary Table 2), OR of predictor, 95% CI of OR, p value.

|  | **Odds of coughing** | | |
| --- | --- | --- | --- |
| *Predictors* | *Odds ratio* | *CI* | *p* |
| (Intercept) | 0.17 | 0.06 – 0.47 | **0.001** |
| Time using LPG per week: 0.1-64.9% | 0.82 | 0.53 – 1.26 | 0.357 |
| Time using LPG per week: 65-99.9% | 0.77 | 0.48 – 1.25 | 0.289 |
| Time using LPG per week: 100% | 0.61 | 0.35 – 1.06 | 0.080 |
| Age | 0.99 | 0.98 – 1.01 | 0.281 |
| Education level: Primary school | 0.94 | 0.45 – 1.96 | 0.862 |
| Education level: Secondary school | 0.97 | 0.47 – 1.99 | 0.932 |
| Education level: University | 0.64 | 0.27 – 1.54 | 0.324 |
| Financial security: Definitely not financially secure | 0.94 | 0.54 – 1.64 | 0.824 |
| Financial security: Not quite financially secure | 1.72 | 1.10 – 2.69 | **0.017** |
| Other smokers in the household | 1.52 | 0.89 – 2.61 | 0.128 |
| Chronic health condition* | 1.78 | 1.19 – 2.65 | **0.005** |
| No electricity connection | 1.47 | 1.04 – 2.08 | **0.028** |

**Supplementary Table 17 -** Effect of fuel use behaviour on prevalence of shortness of breath, comparing self-reported time spent cooking using LPG as a percentage of total cooking time (reference = 0% time spent using LPG). Data is presented as odds ratio (OR) with 95% confidence intervals (95% CI) obtained from a multivariate Poisson logistic regression adjusting for confounding variables. Columns from left to right; Predictor (see Supplementary Table 2), OR of predictor, 95% CI of OR, p value.

|  | **Odds of having shortness of breath** | | |
| --- | --- | --- | --- |
| *Predictors* | *Odds ratio* | *CI* | *p* |
| (Intercept) | 0.06 | 0.02 – 0.19 | **<0.001** |
| Time using LPG per week: 0.1-64.9% | 0.82 | 0.59 – 1.60 | 0.911 |
| Time using LPG per week: 65-99.9% | 1.46 | 0.91 – 2.33 | 0.117 |
| Time using LPG per week: 100% | 1.42 | 0.86 – 2.35 | 0.171 |
| Age | 1.00 | 0.99 – 1.02 | 0.878 |
| Education level: Primary school | 0.76 | 0.32 – 1.81 | 0.533 |
| Education level: Secondary school | 1.08 | 0.47 – 2.48 | 0.849 |
| Education level: University | 0.79 | 0.31 – 2.04 | 0.626 |
| Financial security: Definitely not financially secure | 1.09 | 0.57 – 2.09 | 0.792 |
| Financial security: Not quite financially secure | 2.29 | 1.37 – 3.83 | **0.002** |
| Other smokers in the household | 1.25 | 0.65 – 2.42 | 0.498 |
| Chronic health condition* | 1.95 | 1.28 – 2.98 | **0.002** |
| No electricity connection | 1.79 | 1.20 – 2.67 | **0.004** |

**Supplementary Table 18 -** Effect of fuel use behaviour on prevalence of wheezing, comparing self-reported time spent cooking using LPG as a percentage of total cooking time (reference = 0% time spent using LPG). Data is presented as odds ratio (OR) with 95% confidence intervals (95% CI) obtained from a multivariate Poisson logistic regression adjusting for confounding variables. Columns from left to right; Predictor (see Supplementary Table 2), OR of predictor, 95% CI of OR, p value.

|  | **Odds of wheezing** | | |
| --- | --- | --- | --- |
| *Predictors* | *Odds ratio* | *CI* | *p* |
| (Intercept) | 0.05 | 0.01 – 0.20 | **<0.001** |
| Time using LPG per week: 0.1-64.9% | 1.07 | 0.67 – 1.72 | 0.775 |
| Time using LPG per week: 65-99.9% | 0.91 | 0.53 – 1.58 | 0.743 |
| Time using LPG per week: 100% | 0.72 | 0.37 – 1.39 | 0.327 |
| Age | 0.99 | 0.98 – 1.01 | 0.415 |
| Education level: Primary school | 1.82 | 0.55 – 6.03 | 0.329 |
| Education level: Secondary school | 1.95 | 0.60 – 6.37 | 0.266 |
| Education level: University | 1.01 | 0.26 – 3.87 | 0.990 |
| Financial security: Definitely not financially secure | 1.43 | 0.77 – 2.64 | 0.255 |
| Financial security: Not quite financially secure | 1.66 | 0.95 – 2.89 | 0.076 |
| Other smokers in the household | 2.09 | 1.21 – 3.62 | **0.008** |
| Chronic health condition* | 1.32 | 0.79 – 2.21 | 0.293 |
| No electricity connection | 2.08 | 1.42 – 3.06 | **<0.001** |
|  |  |  |  |

**Supplementary Table 19 -** Effect of fuel use behaviour on prevalence of chest tightness, comparing self-reported time spent cooking using LPG as a percentage of total cooking time (reference = 0% time spent using LPG). Data is presented as odds ratio (OR) with 95% confidence intervals (95% CI) obtained from a multivariate Poisson logistic regression adjusting for confounding variables. Columns from left to right; Predictor (see Supplementary Table 2), OR of predictor, 95% CI of OR, p value.

|  | **Odds of having chest tightness** | | |
| --- | --- | --- | --- |
| *Predictors* | *Odds ratio* | *CI* | *p* |
| (Intercept) | 0.08 | 0.02 – 0.38 | **0.002** |
| Time using LPG per week: 0.1-64.9% | 1.07 | 0.66 – 1.73 | 0.778 |
| Time using LPG per week: 65-99.9% | 1.27 | 0.77 – 2.09 | 0.342 |
| Time using LPG per week: 100% | 0.98 | 0.48 – 2.00 | 0.961 |
| Age | 0.99 | 0.97 – 1.01 | 0.318 |
| Education level: Primary school | 0.92 | 0.27 – 3.11 | 0.890 |
| Education level: Secondary school | 1.30 | 0.40 – 4.28 | 0.661 |
| Education level: University | 0.39 | 0.10 – 1.59 | 0.190 |
| Financial security: Definitely not financially secure | 0.95 | 0.51 – 1.75 | 0.863 |
| Financial security: Not quite financially secure | 1.21 | 0.72 – 2.06 | 0.474 |
| Other smokers in the household | 1.86 | 1.07 – 3.22 | **0.027** |
| Chronic health condition* | 2.01 | 1.28 – 3.16 | **0.003** |
| No electricity connection | 2.01 | 1.36 – 2.97 | **<0.001** |

**Supplementary Table 20 -** Effect of fuel use behaviour on prevalence of headaches, comparing self-reported time spent cooking using LPG as a percentage of total cooking time (reference = 0% time spent using LPG). Data is presented as odds ratio (OR) with 95% confidence intervals (95% CI) obtained from a multivariate Poisson logistic regression adjusting for confounding variables. Columns from left to right; Predictor (see Supplementary Table 2), OR of predictor, 95% CI of OR, p value.

|  | **Odds of having headaches** | | |
| --- | --- | --- | --- |
| *Predictors* | *Odds ratio* | *CI* | *p* |
| (Intercept) | 0.54 | 0.31 – 0.94 | **0.030** |
| Time using LPG per week: 0.1-64.9% | 1.10 | 0.89 – 1.36 | 0.396 |
| Time using LPG per week: 65-99.9% | 0.92 | 0.72 – 1.18 | 0.522 |
| Time using LPG per week: 100% | 0.85 | 0.66 – 1.10 | 0.213 |
| Age | 1.00 | 0.99 – 1.01 | 0.510 |
| Education level: Primary school | 1.36 | 0.88 – 2.10 | 0.164 |
| Education level: Secondary school | 1.25 | 0.82 – 1.93 | 0.300 |
| Education level: University | 1.19 | 0.74 – 1.91 | 0.481 |
| Financial security: Definitely not financially secure | 1.00 | 0.77 – 1.28 | 0.980 |
| Financial security: Not quite financially secure | 1.06 | 0.85 – 1.31 | 0.623 |
| Other smokers in the household | 0.93 | 0.66 – 1.31 | 0.678 |
| Chronic health condition* | 1.16 | 0.92 – 1.47 | 0.210 |
| No electricity connection | 1.20 | 0.99 – 1.46 | 0.062 |
|  |  |  |  |

**Supplementary Table 21 -** Effect of fuel use behaviour on prevalence of eye irritation, comparing self-reported time spent cooking using LPG as a percentage of total cooking time (reference = 0% time spent using LPG). Data is presented as odds ratio (OR) with 95% confidence intervals (95% CI) obtained from a multivariate Poisson logistic regression adjusting for confounding variables. Columns from left to right; Predictor (see Supplementary Table 2), OR of predictor, 95% CI of OR, p value.

|  | **Odds of having eye irritation** | | |
| --- | --- | --- | --- |
| *Predictors* | *Odds ratio* | *CI* | *p* |
| (Intercept) | 0.14 | 0.05 – 0.36 | **<0.001** |
| Time using LPG per week: 0.1-64.9% | 0.86 | 0.64 – 1.15 | 0.305 |
| Time using LPG per week: 65-99.9% | 0.74 | 0.52 – 1.04 | 0.087 |
| Time using LPG per week: 100% | 0.43 | 0.26 – 0.72 | **0.001** |
| Age | 1.01 | 1.00 – 1.02 | 0.181 |
| Education level: Primary school | 1.36 | 0.69 – 2.69 | 0.369 |
| Education level: Secondary school | 1.24 | 0.63 – 2.44 | 0.527 |
| Education level: University | 1.07 | 0.50 – 2.30 | 0.859 |
| Financial security: Definitely not financially secure | 1.46 | 0.98 – 2.17 | 0.064 |
| Financial security: Not quite financially secure | 1.36 | 0.94 – 1.98 | 0.103 |
| Other smokers in the household | 1.21 | 0.82 – 1.78 | 0.341 |
| Chronic health condition* | 1.40 | 1.04 – 1.88 | **0.028** |
| No electricity connection | 1.44 | 1.13 – 1.83 | **0.003** |
